# Abdominal versus femoral subcutaneous fat gene expression in non-obese insulin resistant individuals

**DOI:** 10.3389/fendo.2025.1684761

**Published:** 2026-01-07

**Authors:** Saif Badran, Shamma Almuraikhy, Maha Alser, Khaled Naja, Najeha Anwardeen, Hanan H. Abunada, Mohamed Badie Ahmed, Salma Jarrar, Ghanem Aljassem, Sara Iskeirjeh, Atalla Hammouda, Suhail A. Doi, Layla Al-Mansoori, Mohamed A. Elrayess

**Affiliations:** 1 Division of Plastic Surgery, Department of Surgery, Washington University School of Medicine, Saint Louis, MO, United States; 2 Biomedical Research Center, QU Health, Qatar University, Doha, Qatar; 3 Department of Population Medicine, College of Medicine, QU Health, Qatar University, Doha, Qatar; 4 College of Medicine, QU Health, Qatar University, Doha, Qatar; 5 Plastic Surgery Department, Hamad General Hospital, Hamad Medical Corporation, Doha, Qatar; 6 College of Medicine and Public Health, Flinders University, Adelaide, WA, Australia

**Keywords:** adipose tissue dysfunction, GATA3, gene expression, insulin resistance, PPAR-γ, subcutaneous adipose tissue

## Abstract

**Introduction:**

Adipose tissue, once viewed primarily as an energy reservoir, is now recognized as an endocrine organ involved in various physiological functions, including energy balance and immune responses. Interestingly, recent insights suggest that not all overweight or obese individuals exhibit insulin resistance, guiding the focus towards depot-specific adipose gene expression and its metabolic implications. This pilot study investigates adipose depot-specific differences in gene expression in abdominal versus femoral subcutaneous adipose tissue (SAT) among non-obese, insulin-sensitive (IS) and insulin-resistant (IR) individuals, aiming to elucidate underlying mechanisms of metabolic dysfunction.

**Methods:**

Seventeen non-obese participants scheduled for body contouring surgery provided paired SAT biopsies from abdominal and femoral regions. Based on HOMA-IR scores, participants were categorized into IS and IR groups. Gene expression of key markers including GATA3, PPAR-γ, adiponectin, PGC-1α, TNF-α, and IL-6 were analyzed using real-time PCR and Western Blot. Vitamin D levels were also measured.

**Results:**

Our analysis revealed a complex depot-specific regulation of GATA3 expression in human subcutaneous adipose tissue. While GATA3 protein showed a trend toward higher expression in abdominal SAT, its mRNA levels were significantly elevated in thigh SAT exclusively in insulin-resistant individuals. Furthermore, circulating vitamin D levels correlated significantly with GATA3 and PPARγ expression specifically in thigh adipose tissue.

**Conclusion:**

This pilot study reveals new evidence that the functional differences between abdominal and thigh subcutaneous adipose tissue also encompass transcriptional and post-transcriptional regulation of key adipogenic factors.

## 1 Introduction

The escalation of obesity to pandemic levels now impacts people of all ages worldwide. The World Health Organization reports that, over the past five decades, the prevalence of obesity has increased threefold, highlighting the urgent need for effective public health interventions. (1). This escalation has led to a significant increase in metabolic diseases such as insulin resistance (IR), type 2 diabetes mellitus (T2D), and cardiovascular diseases. Historically, fat depots were regarded merely as storage for excess energy. However, this perspective has shifted to recognize fat as an active endocrine organ that secretes several hormones, known as adipokines, which play a critical role in metabolism, particularly in glucose homeostasis and IR. The latter is defined as a suboptimal bodily response to insulin, resulting in elevated blood glucose levels and eventually leading to T2D (2). However, recent studies have indicated that not all obese individuals are insulin resistant (3). Indeed, there is a subset of overweight and obese individuals who appear to be protected against obesity-related IR and are considered metabolically healthy. This discovery has driven research into the mechanisms linking obesity and IR.

Various studies have examined the impact of fat distribution on IR. Visceral adipose tissue (VAT), which constitutes approximately 15-20% of total body fat, has been implicated as a major contributor to IR and metabolic diseases. This is potentially due to its anatomical and physiological differences from subcutaneous adipose tissue (SAT). Anatomically, VAT is more cellular, vascular, immune cell-rich, and contains more glucocorticoid and androgen receptors. Additionally, VAT delivers free fatty acids (FFAs) directly to the liver via the portal circulation. Physiologically, VAT is more metabolically active, more sensitive to adrenergic stimulation, less absorptive, and has a higher capacity to generate FFAs compared to SAT (4). Although SAT is more absorbent and has a lower capacity to generate and deliver FFAs to the liver via systemic circulation, it still plays an essential role in IR and glucose homeostasis. In fact, intrahepatic triglycerides are more strongly correlated with IR than VAT volume. This correlation has been supported by findings on the beneficial impact of surgical removal of SAT during body contouring surgeries on IR, adipokine levels, and inflammation (5).

Investigating the differences between various SAT depots, it has been observed that upper body fat at the abdomen is more strongly linked to IR and metabolic disease than lower body fat in the gluteo-femoral area. Abdominal circumference, which includes abdominal SAT, VAT, and other internal organs, is a key component in diagnosing metabolic syndrome (6). Abdominal SAT is characterized by a higher lipolytic rate, smaller adipocyte size, and higher secretion of inflammatory adipokines and inflammation levels compared to lower body SAT in the gluteo-femoral region (7). Understanding these depot-specific differences could lead to more targeted and innovative treatment approaches. Several studies have focused on the impact of adipocyte function rather than total volume. Research indicates a stronger correlation between adipocyte dysfunction and insulin resistance as opposed to total body weight or fat mass (8).

Different mechanisms have been examined to understand the relationship between adipocyte dysfunction and IR. There is growing evidence from both animal and human studies suggesting a strong correlation and causative relationship between SAT inflammation and macrophage infiltration as major contributors to obesity-associated IR and metabolic disease (9, 10). This has been supported by contemporary pharmacogenetic studies attenuating obesity-induced IR by reducing adipocyte inflammation (11). The mechanistic pathway behind obesity-induced adipocyte inflammation appears to be cell necrosis (12), but the links between obesity, inflammation, and IR remain under investigation. Recent studies have highlighted the potential role of inflammatory markers, particularly interleukin-6 (IL-6), in the pathogenic role of SAT in IR among the obese population (13). Another emerging area of interest is the role of impaired adipogenesis and related mediators in the development of IR and type 2 DM. Notably, Peroxisome proliferator-activated receptor gamma (PPAR-γ) and GATA3 play crucial roles in this process. PPAR-γ is crucial for adipocyte differentiation and lipid metabolism, and its expression is often associated with improved insulin sensitivity. Conversely, GATA3 inhibits PPAR-γ expression, adipogenesis, and is associated with IR (14). In contrast, GATA3 suppresses PPAR-γ, inhibits adipogenesis, and has been implicated in insulin resistance. Additionally, vitamin D has been shown to influence adipocyte biology by regulating adipogenesis, adipokine secretion, and inflammatory signaling—processes that are critical for maintaining healthy adipose tissue function and overall metabolic homeostasis (15).

This pilot study investigates SAT depot-specific (abdominal versus femoral SAT fat depot) gene expression related to adipocyte inflammation, impaired adipogenesis, and Vitamin D in the non-obese population, and examines whether these differences vary between insulin-sensitive versus insulin-resistant populations. This, in turn, will shed light on potential depot-specific mechanisms linking IR and SAT dysfunction.

## 2 Methods

### 2.1 Cohort and samples collection

A cohort of seventeen non-obese individuals (lean and overweight), who were scheduled for body contouring surgery at Hamad General Hospital (HGH), participated in this study which was conducted between July 2022 and December 2023. Participants were excluded if they had a BMI ≥ 30 Kg/m², experienced unstable weight (defined as a change of ≥5% in body weight) in the past six months, were diabetic or on diabetes medication, or had undergone obesity surgery within the previous two years. Written informed consent was obtained from all participants prior to surgery. During surgery, two SAT biopsies (1 gram each) were collected from the abdominal and thigh areas. The samples were then transported on ice to Qatar University for same-day analysis.

### 2.2 Serum analysis

Baseline blood measurements, including fasting plasma glucose (FPG), lipid profile, and liver function enzymes, were conducted at HGH using a standard chemistry analyzer (Cobas; Roche Diagnostics, Mannheim, Germany). IR was evaluated using the Homeostatic Model Assessment for Insulin Resistance (HOMA-IR), with a cut-off value of 2 used to define insulin resistance (16).

### 2.3 Gene expression assessment

The gene expression assessment was conducted on thigh and abdominal SAT samples at the RNA level by real time PCR method and at the protein levels by Western Blot. From each sample, 20 mg of frozen tissue were cut and physically homogenized with a tissue grinder and pastel. Then RNA was extracted using the Qiazol lysis reagent (Qiagen, 79306) following the TRizol general method according to the manufacturer’s instructions. 1700 ng of total RNA were used for first-strand cDNA synthesis using the High capacity cDNA synthesis kit (applied biosystems, 4368814) following the manufacturer’s protocol. 170 ng of the cDNA product was used as a template for the Real-time PCR using the Luna^®^ Universal qPCR Master Mix (New England Biolabs, M3003E) following the manufacturer’s instructions. We assessed the genes of interest expression using the specific primers (**Supplementary Table S1**) using the 7500 Real-Time PCR System from Applied Biosystem following the PCR conditions recommended by the kit. Real-time PCR was conducted in triplicate (technical replicates), and the GAPDH was used as a housekeeping gene for normalization of the amplified signals of the target genes. The data analysis was performed using the ΔΔCT-based calculations.

For Western Blotting, whole protein lysates were extracted from human tissues. Samples were collected from abdominal and thigh IR and IS subjects. The tissue samples were sonicated and homogenized using cold Radioimmunoprecipitation Assay (RIPA) buffer (~300 µL/50mg tissue) supplemented with protease and phosphatase inhibitors (Thermo Fisher Scientific, Waltham, MA, USA; catalog # 89900, A32963, A32957). Then incubated on ice for 40 min and centrifuged for 20 min at 4°C at 15,000 RPM. The supernatant’s proteins were collected and concentration was measured using bicinchoninic acid (BCA) protein assay (Thermo Fisher Scientific, catalog # 23225). Protein lysates (80 µg) were loaded to 10% sodium dodecyl sulphate (SDS) polyacrylamide gels and subjected to gel electrophoresis. Afterward, transfer of protein lysates from the SDS gels to polyvinylidene fluoride (PVDF) membranes (Thermo Fisher Scientific, catalog # LC2005) was conducted, next all membranes were blocked at room temperature for 1 h using 5% non-fat dry milk prepared in Tris-buffered saline (TBS) with 0.05% Tween 20 (Tween 20; Bio-Rad, Hercules, CA, USA; catalog # 170653). Then membranes were incubated at 4°C for overnight with GATA3 primary antibody (1:1000, Abcam, Cambridge, UK; catalog # ab199428) and anti-GAPDH antibody (1:1000, Cell Signaling, 2118S) as a loading control. The membranes were washed with TBST for 3 times and then were incubated for 1 h at room temperature with horseradish peroxidase-conjugated anti-rabbit IgG secondary antibody (1:2000, Cell Signaling, 7074S). Then, Enhanced Chemiluminescence (ECL) Substrate (Thermo Fisher Scientific, catalog # 34580) was used to detect the targeted proteins. Finally, blots were imaged by ChemiDoc MP Imaging System (Bio-Rad, Hercules, CA, USA; catalog # 12003154) and were analyzed by Image Lab software (version 6.1) to quantify protein band intensities.

### 2.4 Statistical analysis

The cohort was divided into an insulin-sensitive group (n=10, HOMA IR ≤ 2) and an insulin-resistant (IR) group (n=7, HOMA-IR >2). Since the fat samples were collected from both compartments within the same individuals, inter-subject variability was eliminated. Therefore, a paired t-test was employed to analyze the differences in gene expression between the two fat depots. All comparisons and calculations were performed on matched samples obtained from the same individuals, with samples derived from both the abdominal and thigh regions. This matching helps control for individual-specific variability and confounding factors, such as metabolic characteristics and lifestyle influences. Additionally, we applied False Discovery Rate correction to all relevant correlational analyses and depot-specific comparisons. As this was an observational study, power calculations were not deemed necessary (17). The null hypothesis was of no difference between groups. Exact *p* values are reported and *p*-values of <0.05 were taken to indicate data differences that were statistically divergent from the null hypothesis.

## 3 Results

### 3.1 Participant characteristics

Seventeen individuals undergoing body contouring surgery at HGH were recruited. Participants were dichotomized into IS and IR groups based on their HOMA-IR using 2 as cut-off. Accordingly, 59% were IS and 41% IR. Baseline characteristics revealed no statistically divergent differences in gender distribution or BMI. However, the IR group had a higher HbA1C, lower HDL, and lower sodium compared to the IS group (**Table 1**).

**Table 1:** Participant characteristics.

Variable	IS (n=10)	IR (n=7)	p-value
Gender (M/F)	2/8	2/5	0.99
BMI	25.32 (0.29)	27.04 (2.14)	0.125
HOMA-IR	1.18 (0.27)	3.73 (1.56)	0.005
HbA1C	5.05 (4.93-5.25)	5.5 (5.3-6.05)	0.009
TC	4.48 (0.2)	3.86 (0.76)	0.158
TG	0.71 (0.36)	1 (0.38)	0.099
HDL	1.72 (0.88)	1.24 (0.27)	0.007
LDL	2.46 (3.76)	2.18 (0.51)	0.424
Sodium	140 (140-142)	139 (139-139.5)	0.024
Potassium	4.5 (4.3-4.5)	4.4 (4.25-4.6)	1.000
Chloride	104.67 (1.88)	102.86 (1.86)	0.053
Bicarbonate	25.44 (0.09)	24.71 (2.06)	0.478
Calcium	2.26 (2.24-2.32)	2.27 (2.22-2.46)	0.68
Urea	3.69 (8.46)	4.69 (1.39)	0.129
Creatinine	53 (51-58)	52 (50-63)	1.000
Albumin	37.44 (38.39)	34.51 (10.12)	0.478
Uric acid	259.8 (5.24)	254.71 (18.61)	0.723
ALT	12.78 (5.15)	19.51 (10.18)	0.148
AST	15.5 (13.75-17)	15 (14-17)	0.954
Vit D	26.1 (50.94)	24 (8.91)	0.710
Ferritin	34.95 (17.62-62.68)	45.4 (40.55-49.25)	0.193
TSH	1.7 (3.95)	2.4 (1.66)	0.355

Data are presented as mean (SD)/median (IQR) based on the normality (Shapiro Wilk’s) test results. The parametric/non-parametric measurements between IS vs IR groups were analyzed using Student’s t test/Mann Whitney U test. P-values of <0.05 were taken to indicate data that were statistically divergent from the null hypothesis.

### 3.2 Gene expression differences in IS and IR groups

**Figure 1:**
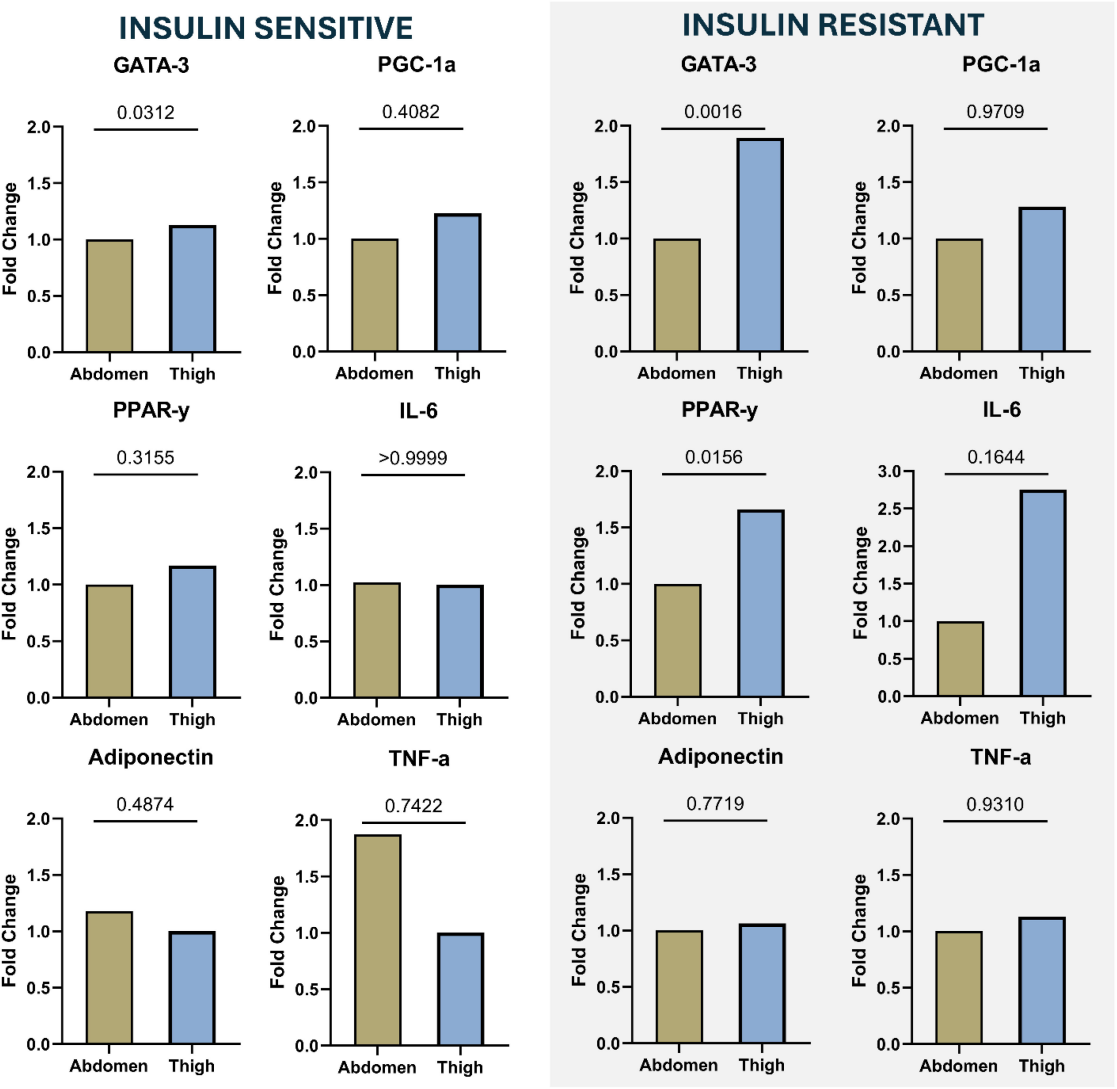
Relative mRNA expression of GATA-3, PGC-1α, PPAR-γ, IL-6, Adiponectin, and TNF-α in abdominal and thigh subcutaneous adipose tissue depots from IS (n=10) and IR (n=7) individuals. mRNA levels were quantified and normalized within each group. Statistical significance is shown above each comparison. Normality test was performed between SAT depots in each groups separately. Outliers were removed using ROUT statistical analysis. P-values of <0.05 were taken to indicate data that were statistically divergent from the null hypothesis.

In the IS group, no statistically divergent differences were observed between abdominal and thigh fat depots regarding the expression levels of GATA3, PPAR-γ, Adiponectin, PGC-1α, TNF-α, and IL-6 (**Figure 1**). However, in the IR group, thigh GATA3 levels were found to be twice as high as those in the abdomen, and thigh PPAR-γ levels were 1.5 times higher than those in the abdomen.

**Figure 2:**
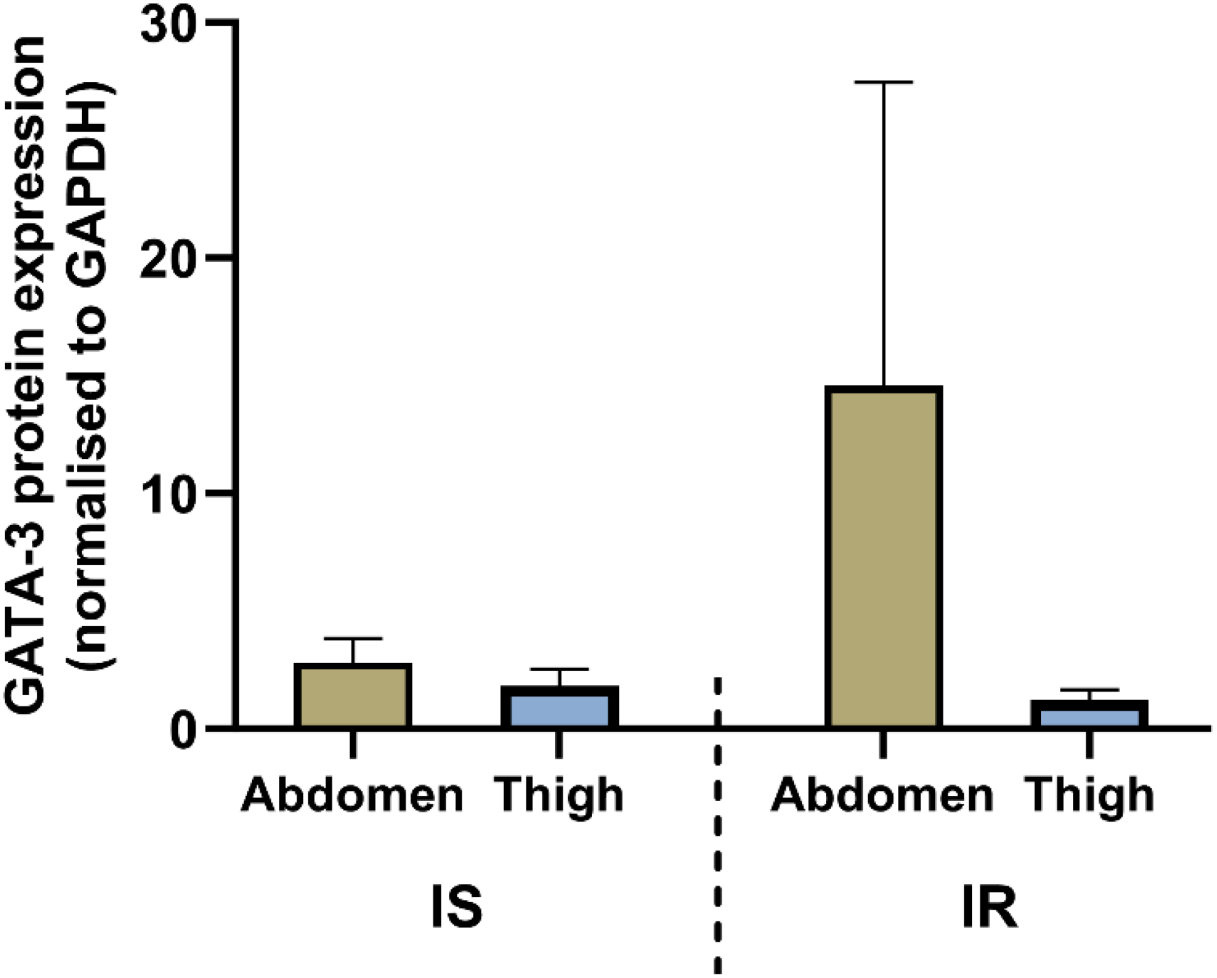
GATA3 protein expression in abdominal and thigh SAT in IR (n=5) and IS (n=4) groups. Protein levels were quantified by Western blotting and represent pooled densitometry values normalized to GAPDH. Data are expressed as mean ± SEM. P-values of <0.05 were taken to indicate data that were statistically divergent from the null hypothesis.

In contrast, Western blot data (**Supplementary Figure S1**) reveals that GATA3 protein expression was consistently lower in thigh adipose tissue compared to the abdomen across both insulin-sensitive and insulin-resistant groups (**Figure 2**).

### 3.3 Vitamin D correlation with gene expression in thigh and abdominal SAT

Exploring depot-specific correlations between vitamin D levels and gene expression may provide insights into mechanisms underlying metabolic risk in non-obese individuals.

**Figure 3:**
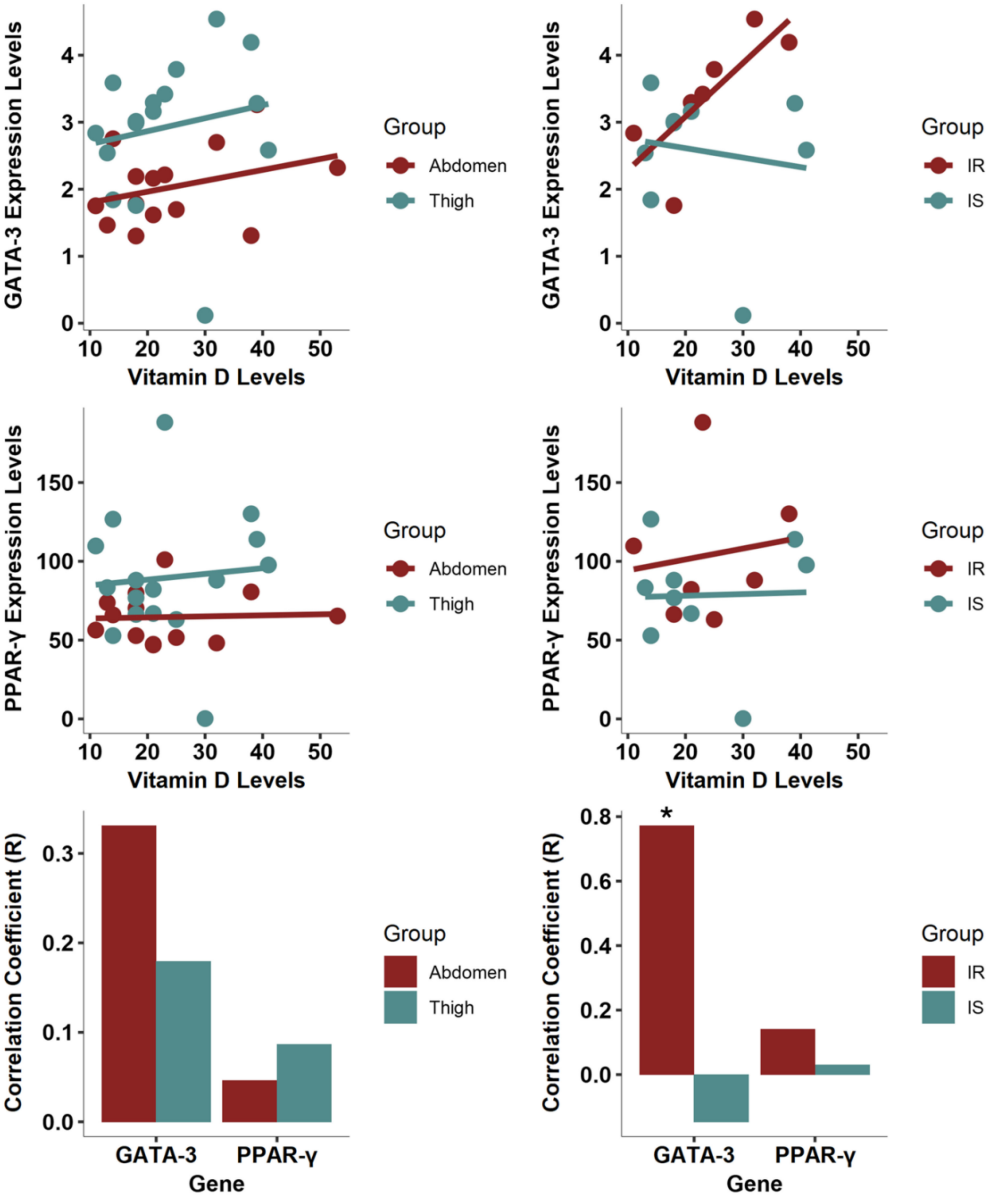
Vitamin D correlation with gene expression in thigh and abdominal SAT. Left Panel: Correlation across different depots, with lines indicating trends for each depot. Right Panel: Correlation in thigh tissue only, showing the trends in IS and IR group. * Indicates statistical divergence was observed.

Pearson correlation analysis was performed between serum vitamin D levels and the measured gene expression levels of GATA3, PPAR-γ, Adiponectin, PGC-1α, TNF-α, and IL-6 (**Figure 3**). A statistically divergent correlation was identified between vitamin D and thigh levels of GATA3 (R = 0.61, p-value 0.01) and PPAR-γ (R = 0.60, p-value 0.02), but not for abdominal levels. When segregating the groups based on their HOMA-IR index, the correlation between vitamin D and thigh levels of GATA3 and PPAR-γ remained strong in the IS group. In contrast, the IR group exhibited a correlation only between vitamin D and thigh GATA3 levels.

## 4 Discussion

Both visceral and subcutaneous fat play critical roles in the pathogenesis of insulin resistance (18). Adipose tissue depots exhibit significant metabolic heterogeneity. Upper body fat, particularly visceral and subcutaneous abdominal fat, is typically associated with a higher risk of metabolic dysfunction. In contrast, lower body fat, especially in the thighs, is often viewed as a protection against metabolic diseases (19). This supports the hypothesis that thigh fat depots differ from abdominal fat in terms of gene expression and metabolic outcomes. Different mechanisms have been examined to understand the relationship between adipocyte dysfunction and IR, including impaired adipogenesis (20) and adipocytes inflammation (21). However, SAT fat depot functional heterogeneity have not been fully understood. This study investigates gene expression differences between abdominal and thigh SAT in non-obese individuals (BMI < 30) in relation to impaired adipogenesis and inflammation, and examines whether these differences vary between insulin-sensitive versus insulin-resistant populations.

Our results reveal a complex, depot-specific regulation of GATA3 in human subcutaneous adipose tissue, modulated by systemic insulin sensitivity. Although Western blot analysis did not reveal statistically significant differences, most likely due to the small sample size, there was a clear trend toward higher GATA3 protein expression in abdominal SAT compared with thigh SAT. This observation is in line with our previous data (22) showing that GATA3 protein expression tends to be greater in abdominal fat depots, a region known to be more metabolically active and less insulin-sensitive compared with gluteofemoral fat (23).

In contrast, the mRNA expression results demonstrated a distinct pattern, with significantly higher GATA3 transcript levels in thigh SAT compared to abdominal fat, but only in insulin-resistant individuals. This discrepancy between mRNA and protein levels suggests a post-transcriptional regulatory mechanism, where GATA3 transcript abundance does not directly translate into corresponding protein accumulation. We hypothesize here that depot-specific differences in the cellular microenvironment may underpin this divergence, and the distinct lipid composition of these depots provides a plausible explanation. For instance, abdominal SAT, shown to be enriched in saturated fatty acids (24), may foster an environment that promotes GATA3 protein stability. Conversely, the polyunsaturated fatty acid rich milieu of thigh SAT (24) might enhance transcriptional activity or mRNA stability but not support equivalent protein accumulation, possibly by modulating signaling pathways that affect translation (25). Additionally, abdominal subcutaneous depots are demonstrated to have smaller adipocytes compared with femoral/thigh adipose tissue (26), which can affect rates of protein synthesis, turnover, and the relative contribution of stromal vascular cells to measured signals. In other words, smaller adipocytes and/or a different stromal cell composition in abdominal fat could create a microenvironment where GATA3 protein is more readily retained or stabilized, while larger or more lipid-loaded thigh adipocytes might show more transcriptional responsiveness but faster protein turnover. Notably, when we compared gene expression between IS and IR individuals (**Supplementary Table S2**), we did not find any statistically significant differences for the key markers studied. This indicates that systemic insulin resistance alone, in the absence of a depot-specific context, is not a primary driver of divergent expression for these genes. **Supplementary Table S3** also shows the correlation of HOMA-IR with gene expression in abdominal and thigh adipose tissue depots.

This transcriptional adaptation in thigh fat appears to be part of a coordinated program, as PPARγ mRNA was similarly upregulated in the IR group. Given that GATA3 and PPARγ exert opposing effects on adipogenesis (27), their parallel induction may reflect a homeostatic mechanism to maintain adipocyte function in the lower-body depot under insulin-resistant conditions. This could serve to enhance the lipid-buffering capacity of thigh fat, thereby limiting ectopic lipid spillover.

The significant correlation between vitamin D levels and the expression of GATA3 and PPAR-γ in thigh fat suggests a possible regulatory role of vitamin D in adipogenesis and insulin sensitivity.

Vitamin D’s involvement in various metabolic processes, paired with its deficiency often being linked to insulin resistance and obesity (28), indicates that vitamin D might differentially affect metabolic roles and receptor expression in these fat depots. Our study design involved matching samples from the abdomen and thighs of the same individuals, which minimizes the potential confounding effects of other metabolic factors such as dietary intake, sun exposure, and calcium levels. Despite this control, we acknowledge that our findings do not establish causality between vitamin D levels and the expression of GATA3 and PPAR-γ. Further mechanistic studies are necessary to establish a causal relationship and to explore the influence of additional lifestyle and metabolic factors.

This pilot study boasts several strengths, including a comparative analysis of gene expression in two distinct fat depots (abdominal and thigh SAT) in a non-obese population. By examining key adipogenic and inflammatory markers in both depots from the same individuals, excluding obese and diabetic patients, and separating the cohort into IS and IR groups, the analysis is strengthened by eliminating other potential confounding factors that could affect the comparison. However, the study also has several limitations. The relatively small sample size may limit the generalizability of the results. Additionally, the study focuses solely on non-obese individuals, which may not capture the full spectrum of metabolic variations observed in obese populations. Moreover, we were unable to evaluate PPARγ protein expression because of technical challenges. Future studies should aim to include larger, more diverse populations to enhance the generalizability of the results. Research should also explore the impact of various lifestyle and genetic factors on fat depot-specific gene expression and metabolic outcomes. Investigating the mechanisms through which vitamin D influences gene expression in different fat depots can further elucidate its role in metabolic health. Additionally, longitudinal studies that track changes over time and assess the effects of interventions targeting specific fat depots would provide deeper understanding and potentially inform more targeted therapeutic approaches for metabolic disorders. Moreover, the proposed mechanistic relationship between the depot-specific fatty acid microenvironment and the post-transcriptional regulation of GATA3 protein stability and turnover remains to be experimentally confirmed.

## 5 Conclusion

This pilot study provides novel evidence that the functional heterogeneity between abdominal and thigh subcutaneous adipose tissue extends to the transcriptional and post-transcriptional regulation of key adipogenic factors. We identified a clear discordance in the depot-specific expression of GATA3, characterized by higher protein levels in abdominal SAT but elevated mRNA levels in the thigh SAT of insulin-resistant individuals. This suggests that the metabolic properties of these depots are shaped not only by transcriptional activity but also by potent post-transcriptional mechanisms, potentially influenced by their distinct lipid compositions and cellular environments.

The coordinated upregulation of the antagonistic regulators GATA3 and PPARγ in thigh fat during insulin resistance points to a complex, adaptive response aimed at maintaining adipocyte function and systemic lipid homeostasis. Furthermore, the correlation of these factors with vitamin D levels highlights a potential modifiable influence on thigh fat biology. Understanding these depot-specific adaptive mechanisms may reveal new therapeutic targets for managing metabolic disease by harnessing the beneficial properties of lower-body adipose tissue.

## Data Availability

The raw data supporting the conclusions of this article will be made available by the authors, without undue reservation.

## Supplementary material

Supplementary Table 1 List of forward and reverse primers used for gene expression analysis.

Supplementary Table 2 Relative mRNA expression of GATA-3, PGC-1α, PPAR-γ, IL-6, Adiponectin, and TNF-α in Insulin-Sensitive (IS; n=10) and Insulin-Resistant (IR; n=7) Individuals Across Both Adipose Tissue Depots. mRNA levels were quantified and normalized within each group.

Supplementary Table 3 Correlation Between HOMA-IR and Gene Expression in Abdominal (n=17) and Thigh (n=17) Adipose Tissue Depots.

Supplementary Figure 1 Western blot analysis of GATA3 protein expression in paired abdominal (A) and thigh (T) adipose tissue samples from insulin-sensitive (IS) and insulin-resistant (IR) groups. Protein abundance was normalized to GAPDH as a loading control. The molecular weight marker (PM2500) is displayed on the right for reference.
