# Supplementary material for: Abdominal versus femoral subcutaneous fat gene expression in non-obese insulin resistant individuals
Source: Front Endocrinol (Lausanne). 2026 Jan 7;16:1684761. doi: 10.3389/fendo.2025.1684761 (PMC12819261; doi:10.3389/fendo.2025.1684761)
Supplement: Supplementary Table 1 — List of forward and reverse primers used for gene expression analysis. [file DataSheet1.docx]

**Supplementary Table 1.** List of forward and reverse primers used for gene expression analysis.

| Gene | Primers sequences (5′ to 3′) |
| --- | --- |
| h-GAPDH | *f:* CGA CAG TCA GCC GCA TCTT |
|  | *r:*  CCG TTG ACT CCG ACC TTCA |
| h-GATA3 | *f:* ACCACAACCACACTCTGGAGGA |
|  | *r:* TCGGTTTCTGGTCTGGATGCCT |
| h-PPAR-γ | *f:* AGCCTGCGAAAGCCTTTTGGTG |
|  | *r:* GGCTTCACATTCAGCAAACCTGG |
| h-PGC1 - α | *f:* AGCCTCTTTGCCCAGATCTT |
|  | *r:* GGCAATCCGTCTTCATCCAC |
| h-TNF-α | *f:* TGGAGCCTCGAATGTCCATT |
|  | *r:* ACCCCGGCCTTCCAAATAAA |
| h-IL-6 | *f:* ACAAAGCCAGAGTCCTTCAGA |
|  | *r:* ATGGTCTTGGTCCTTAGCCA |

**Supplementary Table 2**. Relative mRNA expression of GATA-3, PGC-1α, PPAR-γ, IL-6, Adiponectin, and TNF-α in Insulin-Sensitive (IS; n=10) and Insulin-Resistant (IR; n=7) Individuals Across Both Adipose Tissue Depots. mRNA levels were quantified and normalized within each group.

|  | IS | IR | p-value |
| --- | --- | --- | --- |
| Gata3 | 1 | 1.1 | 0.65 |
| PPAR-y | 1 | 0.88 | 0.97 |
| Adiponectin | 1 | 0.87 | 0.43 |
| PGC-1a | 1 | 0.87 | 0.70 |
| IL-6 | 1 | 1.56 | 0.56 |
| TNF-a | 1 | 2.4 | 0.82 |

**Supplementary Table 3**. Correlation Between HOMA-IR and Gene Expression in Abdominal (n=17) and Thigh (n=17) Adipose Tissue Depots.

|  | Abdomen | | Thigh | |
| --- | --- | --- | --- | --- |
|  | r | p-value | r | p-value |
| Gata3 | -0.32 | 0.25 | 0.38 | 0.14 |
| PPAR-y | -0.22 | 0.45 | 0.08 | 0.75 |
| Adiponectin | -0.49 | 0.04 | -0.18 | 0.47 |
| PGC-1a | -0.22 | 0.44 | -0.11 | 0.67 |
| IL-6 | -0.04 | 0.87 | 0.34 | 0.22 |
| TNF-a | -0.11 | 0.67 | 0.29 | 0.27 |

**
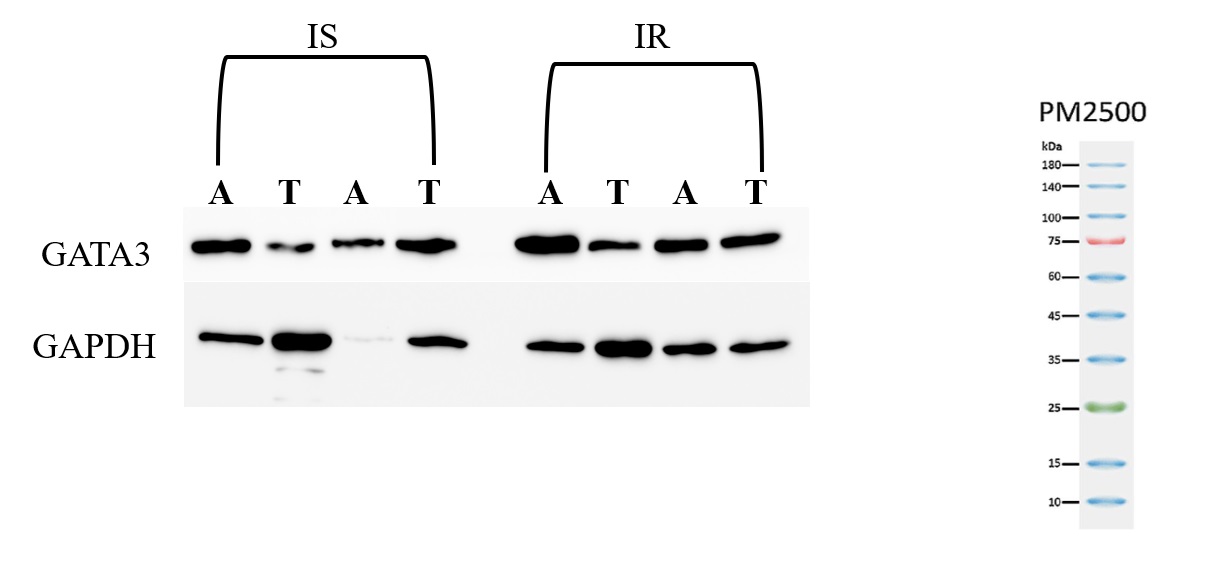
**

**Supplementary figure 1.** Western blot analysis of GATA3 protein expression in paired abdominal (A) and thigh (T) adipose tissue samples from insulin-sensitive (IS) and insulin-resistant (IR) groups. Protein abundance was normalized to GAPDH as a loading control. The molecular weight marker (PM2500) is displayed on the right for reference.
